# Basil Essential Oil: Composition, Antimicrobial Properties, and Microencapsulation to Produce Active Chitosan Films for Food Packaging

**DOI:** 10.3390/foods10010121

**Published:** 2021-01-08

**Authors:** Ghita Amor, Mohammed Sabbah, Lucia Caputo, Mohamed Idbella, Vincenzo De Feo, Raffaele Porta, Taoufiq Fechtali, Gianluigi Mauriello

**Affiliations:** 1 Department of Agricultural Sciences, University of Naples Federico II, Via Università 100, 80055 Portici, Italy; amor.ghitaa@gmail.com (G.A.); mohamed.idbella@usmba.ac.ma (M.I.); 2 Laboratory of Biosciences, Integrated and Molecular Functional Exploration, Faculty of Sciences and Techniques-Mohammedia, University Hassan II 146, Mohammedia 20650, Morocco; toufiqr@yahoo.com; 3 Department of Nutrition and Food Technology, An-Najah National University, Nablus P.O. Box 7, Palestine; m.sabbah@najah.edu; 4 Department of Pharmacy, University of Salerno, Via Giovanni Paolo II 132, 84084 Fisciano, Italy; lcaputo@unisa.it (L.C.); defeo@unisa.it (V.D.F.); 5 Department of Chemical Sciences, University of Naples “Federico II”, 80126 Naples, Italy; raffaele.porta@unina.it

**Keywords:** basil essential oil, microencapsulation, chitosan film, food shelf life, food packaging, cooked ham

## Abstract

The essential oil (EO) from basil—*Ocimum basilicum*—was characterized, microencapsulated by vibration technology, and used to prepare a new type of packaging system designed to extend the food shelf life. The basil essential oil (BEO) chemical composition and antimicrobial activity were analyzed, as well as the morphological and biological properties of the derived BEO microcapsules (BEOMC). Analysis of BEO by gas chromatography demonstrated that the main component was linalool, whereas the study of its antimicrobial activity showed a significant inhibitory effect against all the microorganisms tested, mostly Gram-positive bacteria. Moreover, the prepared BEOMC showed a spheroidal shape and retained the EO antimicrobial activity. Finally, chitosan-based edible films were produced, grafted with BEOMC, and characterized for their physicochemical and biological properties. Since their effective antimicrobial activity was demonstrated, these films were tested as packaging system by wrapping cooked ham samples during 10 days of storage, with the aim of their possible use to extend the shelf life of the product. It was demonstrated that the obtained active film can both control the bacterial growth of the cooked ham and markedly inhibit the pH increase of the packaged food.

## 1. Introduction

Food industry is developing new packaging materials, even by the incorporation of volatile antimicrobial agents, such as essential oils (EOs), into the polymeric films [1]. EOs, obtained from different plant organs (flowers, buds, seeds, leaves, twigs, etc.), are complex mixtures of volatiles compounds endowed with antimicrobial and antifungal, as well as antioxidant properties [2]. Therefore, their addition to packaging materials can lead to incorporation of their components into the food, neutralizing spoilage microorganisms present in the packaged food and extending product shelf life [3,4]. In particular, the EOs of the different cultivars of basil (*Ocimum basilicum* L.) (BEO) have been shown to possess analgesic, anti-inflammatory, antibacterial, hepatoprotective, and immunomodulatory properties [5]. In fact, BEO is sometimes used as an additive to avoid food oxidation or as an antimicrobial agent, or as an ingredient to affect the flavor and aroma of different products [6,7]. The microencapsulation is one of the most effective new techniques for protecting compounds against volatilization, oxidation, and thermal degradation [8,9]. Various methods are employed to form microcapsules, the most remarkable of which are spray drying, spray air suspension coating, centrifugal extrusion, centrifugal suspension–separation and vibration technology [10]. Vibration technology, generally used for the production of microspheres and microcapsules, consists of breaking up a laminar liquid stream into droplets by a superimposed vibration. This technique has gained a significant interest in the last years mainly due to the possibility to produce very uniform monodisperse microcapsules [11] and represents a potentially significant growth area in food industry [12].

Microencapsulation of EOs in edible films and coatings has been advocated as a “natural” alternative procedure to the addition of chemical, and often potentially toxic, antimicrobial agents to food packaging materials [13]. In addition, it has a great prospective in the industry due to its capability to transform conventional polymers or biopolymers into intelligent and multifunctional materials useful for food preservation. In particular, chitosan (CH) edible films containing microcapsules of EOs may be an innovative packaging system to extend the commercial shelf-life of various food products, avoiding the addition of chemical preservatives [14]. Although numerous studies on the antimicrobial effectiveness of free and microencapsulated EOs are available [15,16,17], very few data are available on EO microencapsulation by vibration technology in packaged foods. The aim of this work, thus, was to evaluate the effectiveness of an innovative active CH-based packaging system containing BEO microcapsules to extend the shelf life of cooked ham.

## 2. Materials and Methods

### 2.1. Chemicals and Microorganisms

BEO was purchased from Sinergy Flavors Italy S.p.A. (Trieste, Italy). BEO was extracted by hydro distillation as reported in the technical sheet of the product. CH (molar mass 3.7 × 10^4^ g/mol, 91% N-deacetylation) was a gift from prof. R.A.A. Muzzarelli (University of Ancona, Italy) and characterized as previously described [18]. All further chemicals and solvents were analytical grade and are cited with supplier and code in bracket. Gram-positive and Gram-negative bacteria reported in Table 1 were from the culture collections of the Department of Agricultural Sciences, University of Naples Federico II. All of them were previously identified at genome level by 16S rRNA gene sequencing. Microbial strains were routinely grown in tryptone soya broth (TSB, Thermo Fisher Scientific, Rodano, Italy) for 24 h.

### 2.2. GC-FID Analysis of BEO

The GC-FID analysis of basil EO was performed with a gas chromatograph Pekin Elmer Sigma-115 equipped with a flame ionization detector and HP-5 MS fused silica capillary column (30 m × 0.25 mm × 0.25 mm film thickness). The detector and injector temperatures were 250 °C and 290 °C and the injection modes spitless was 1 mL of a 1:1000 n-hexane. Analysis was also run by using a fused silica HP Innowax capillary column (50 m × 0.20 mm, 0.25 mm film thickness). In both cases, the carrier gas was helium at flow rate of (1.0 mL/min).

### 2.3. GC/MS Identification of Single Constituents of BEO

The composition of volatile constituents of basil EO was analyzed by Agilent 6850 Ser. Equipped with MSD 5973 mass selective spectrometer (ionization energy 70 Ev, capillary column 30 m × 0.25 mm × 0.33 film thickness, electron voltage energy 2000 V. The samples were injected as mentioned above and the Injector was heated to a temperature of 295°.The identification of major constituents was achieved by comparing their retention indices relative to C10–C35 n-alkanes with either those of the literature [19,20,21], through mass spectra analysis on both columns and by their comparison with those of the authentic compounds available in our laboratories by means of NIST 02 and Wiley 275 libraries [22]. Finally, components’ relative concentrations were obtained by peak area normalization.

### 2.4. Antimicrobial Activity of BEO

The antimicrobial activity of BEO was tested against the different indicator strains reported in Table 1 by the filter paper disc diffusion method [23]. In particular, 0.1 mL of an overnight culture of each indicator strain with 10^7^ colony forming units (CFU)/mL was spread directly on Tryptone Soy Agar (TSA); TSB with addition of 7.5 g/L agar and yeast extract (Agar bacteriological n.1, Oxoid). Sterile filter paper discs (6 mm in diameter) were first soaked with 20 µL of BEO and then placed on TSA. Finally, all plates were incubated at optimal growth condition culture of each indicator strain for 24 h. The inhibition zones were measured with a caliper and recorded in mm. All tests were performed in triplicates.

The minimum inhibitory concentration (MIC) and the minimum lethal concentration (MLC) of BEO were determined only against the microorganisms that exhibited a strong sensitivity in the previous assay. MIC of BEO was determined using broth dilution method. A serial dilution of BEO, ranging from 40 mg/mL to 0.3 mg/mL, was prepared in test tubes containing Tryptone Soy Broth [24]. Each tube was inoculated with the same volume of bacterial suspension adjusted to 10^6^ CFU/mL. MIC values were defined as the lowest concentration of BEO at which the absence of growth was recorded. Controls of medium with either microorganisms or BEO alone were included. BEO MLC was determined by sub culturing 10 μL from the last four wells without visible bacterial growth onto TSA plate. After incubation at optimal growth conditions for 24 h, MLC was defined as the lowest concentration resulting in a negative subculture or giving presence of only one colony after incubation.

Ethanol (code 02483 Sigma-Aldrich, Milan, Italy) was used as negative control; tetracycline (10 μg) and gentamicin (10 μg) were used as positive control.

### 2.5. Microencapsulation of BEO

Basil EO was microencapsulated by vibration technology [25], using the Encapsulator B-395 Pro (BUCHI, Flawil, Switzerland) equipped with the syringe pump and a nozzle diameter of 120 μ. Briefly, the system is based on the extrusion of a laminar jet of a calcium alginate solution subjected to a preset high frequency mechanical vibration, resulting in a controlled break-up of the laminar jet in spherical drops. The fall in a calcium chloride bath of droplets leads to the gelation of the alginate microbeads under the form of calcium alginate. The feeding solution was carried out by mixing 10 mL of BEO, 0.5 mL of Tween 80 emulsifier and 35 mL of alginic acid sodium salt solution (previously degassed and sterilized by autoclaving at 121 °C for 15 min). Then, the prepared mixture (pH 6.8) was loaded in a 50 mL luer-lock syringe and forced into the pulsation chamber to be further extruded through the nozzle. The microencapsulation parameters were adjusted to flow rate, 3 mL/min, vibration frequency, 200 Hz; electrode voltage, 1800. Microcapsules of BEO were obtained by hardening of the droplets in 150 mL of CaCl_2_ solution continuously stirred at 100 rpm. All the process was performed at room temperature. Finally, the obtained suspension was recovered in batch and stored at 4 °C. After separation of microcapsules and the solution, a final volume of 25 mL of microcapsules of BEO were obtained.

The encapsulation efficiency (EE) was determined using the following equation:

(1) EE=m1/m2∗100

where m1 is the amount, expressed in g, of essential oil contained in the microcapsules, and m2 is the total amount, expressed in g, of BEO used. The amount of BEO contained in the microcapsules (m1) was determined using the following equation:

(2) m1= m2−m3

where m3 is the amount, expressed in g, of BEO from aqueous phase collected after microcapsule filtration by solvent extraction. All experiments were carried out in triplicate and results presented are the average values.

Size and morphology of BEO microcapsules (BEOMC) were examined using both a Zeiss light (200× magnification and calibrated micrometer) and scanning electron microscope (SEM-Evo 40, Carl Zeiss, Oberkochen, Germany). Microcapsules from each encapsulation were visualized immediately after the process by optical microscopy, with no special sample preparation. On the contrary, for SEM analysis, microcapsules were initially rinsed three times with MilliQ water (Lichrosalv water for Chromatography) and then 10 µL of each sample were placed on a pin type SEM specimen mount and maintained at 45 °C for 2 h in order to achieve a gentle dehydration of the microcapsules and their fixing. All samples were sputter treated in a metallizer (Agar Sputter Coater) with gold palladium to reach a thickness of coating of 100 Å and then observed by SEM high vacuum mode (EHT, 20.00 Kv).

### 2.6. Antimicrobial Activity of BEOMC

Resting cell experiment was carried out in order to evaluate BEOMC antimicrobial activity. One mL of microcapsules was added to a bacterial suspension to reach a cell concentration of 10^6^ CFU/mL. The viable count of indicator strain was evaluated by plate counting on TSB agar both immediately (T_0_) and after incubation for 1, 2, 3, 4, 5, and 24 h at 4 °C. A cell suspension without microcapsules was used as control. At T_0_ and after 24 h of incubation, an aliquot of each sample was stained using a LIVE/DEAD BackLight Bacterial Viability Kit (Molecular Probes, Eugene, OR, USA) in order to investigate cell membrane damage. Accordingly to the procedure previously described by Ercolini et.al 2006 [26], fluorochrome stock solution (6 μL) was added to 10 μL of each sample and incubated in the dark for 15 min at room temperature. At the end of incubation, samples were observed using a Nikon Eclipse E400 epifluorescence microscope (Nikon, Tokyo, Japan) equipped with a UV lamp and a 100X magnification objective.

### 2.7. CH-Based Film Preparation

A 3% CH film forming solution (FFS) was prepared as previously described [18,27] with some modifications. CH was dissolved in 0.1 M HCl (code 1.13386 Sigma-Aldrich Italy) at room temperature using an overhead stirrer homogenizer (IKA overhead stirrer homogenizer, RW 20. USA) for about 24 h to complete solubilization, then the pH was adjusted to 4.0 by adding 1 M NaOH (code S5881 Sigma-Aldrich Italy). Glycerol (code G7893 Sigma-Aldrich, Italy) was then added (30% *w*/*w* of CH) and stirred for 3 h at room temperature. Where indicated, BEOMC at different concentrations (1, 2, and 3% *w*/*v*) were added and mixed by vortex. Seventy-five milliliters of the final mixture were casted on a 24 × 18 cm² polypropylene sheet.

FFSs were spread by using COATMASTER device model 510 (ERICHSEN GmbH and Co. Hemer, Germany) with spiral size 80 µm, and then were kept for 5 h to dry at room temperature under ventilated cabinet. Films were peeled from the casting surface and stored at 25 °C and 50% RH for further experiments.

### 2.8. CH-Based Film Properties

Films were characterized for their thickness on six different points by using an electronic digital micrometer with sensitivity of 0.001 mm. Film tensile strength (TS), elongation at break (EB), and Young’s modulus (YM), were determined [28] on six specimens of each different films (5 cm gage length, 1 kN load and 5 mm/1 min speed) by using an Instron universal testing instrument model no. 5543A (Instron Engineering Corp., Norwood, MA, USA). Film opacity was determined as previously described by Giosafatto et al. (2019) [29], six times for each film by measuring:

(3) $$\mathrm{Opacity}\;\left({\mathrm{mm}}^{-1}\right)\;=\;A_{600}/x$$

where A_600_ is the absorbance at 600 nm and x is the film thickness (mm).

The assessment of film antimicrobial activity was carried out on *S. saprophyticus* 3S as Gram-positive and *E. coli* 32 as Gram-negative. For each bacterial strain, inoculum from the stock was revived in Tryptone Soy Broth and incubated at 37 °C for 24 h. Thereafter, the bacterial broth was diluted serially till final concentration of 10^6^–10^7^ CFU/mL (colony forming units/mL) is achieved. One milliliter of the diluted culture broth was taken in a test tube and test film of specific dimension was cut and immersed into the culture broth. The tubes were then incubated at 37 °C for 24 h to allow the interaction between the film and the bacteria. Control samples were simultaneously run without film addition. The viable count was evaluated by plate counting on TSA.

### 2.9. Cooked Ham Wrapping

**Figure 1:**
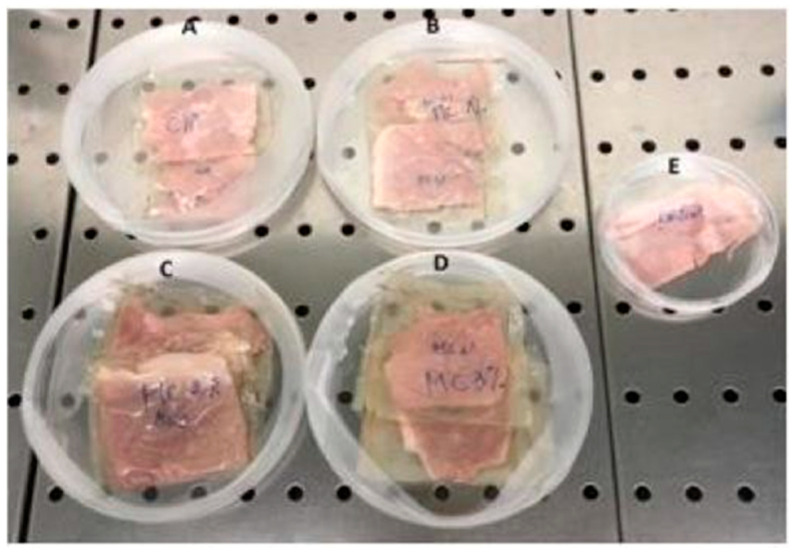
Cooked ham samples wrapped with chitosan films (**A**) containing 1% (**B**), 2% (**C**), or 3% (**D**) basil essential oil microcapsules; control unwrapped cooked ham (**E**).

Cooked ham, obtained from a local supermarket (Naples, Italy), was cut to obtain slices of 10 g and then wrapped with the prepared CH films containing different concentrations of BEOMC. Two control samples were used in this experiment: unwrapped ham and ham wrapped with films prepared with CH alone. Each sample was placed in Petri dishes, as shown in Figure 1, and stored at 4 °C for 10 days.

Cooked ham samples aliquot (10 g) were taken every 2 days during storage and homogenized in a stomacher with 90 mL of sterile buffered peptone water for the determination of total aerobic mesophilic bacteria (AMB) on PCA (Plate Count Agar) incubated at 37 °C for 48 h. Mesophilic lactic acid bacteria (LAB) were determined in MRS agar incubated under anaerobiosis at 37 °C for 72 h. Enterobacteria were determined in VRBGA (Violet Red Bile Glucose Agar) incubated at 37 °C for 24 h. Yeasts were determined in Rose Bengal Agar with chloramphenicol incubated at 28 °C for 3 days. Results were expressed as logarithm of colony forming units per gram of ham. The pH was measured by a HI 221 pH meter (HANNA Instruments, Ronchi di Villafranca Padovana, Italy) and 3 measurements were taken.

### 2.10. Statistical Analysis

John’s Macintosh Project (JMP) software 8.0 (SAS Institute, Cary, NC, USA) was used for all statistical analyses. The data were subjected to analysis of variance (ANOVA), and the means were compared using the student’s-*t* test. Differences were considered to be significant at *p* < 0.05.

## 3. Results and Discussion

### 3.1. BEO Chemical Composition

The detected chemical composition of BEO, reported in Table 2, shows that 52 compounds were identified, and that the component most present by far is linalool (41.3%) followed by 1,8-cineole (9.6%), (Z)-isoeugenol (5.9%), 1-epi-cubenol (4.8%), α-transbergamotene (4.6%), and (Z)-anethol (3.2%). Further compounds, occurring in amounts between 2 and 3%, are trans-muurola-4-(14), 5-diene (2.8%), €-caryophyllene (2.4%), isobornylacetate (2.1%), whereas all the others are present in amounts lower than 2%.

The study of taxonomy of BEO is quite complex because of the numerous botanical varieties, cultivar, and chemotypes [30]. Moreover, a variability due to climatic factors has been described by Milenković et al. (2019) [31], who demonstrated that shade-grown basil plants have a high content of eugenol with respect to plants grown without shading that contain more linalool than eugenol. Olugbade et al. (2017) [32], examined BEO from Sierra Leone and Nigeria; the first was clearly identified as the methyl eugenol chemotype (89.7%), whereas the second was the methyl chavicol (89.8%) chemotype. Moreover, Ghasemi Pirbaoluti et al. (2017) [33], reported that the major constituents of EO extracted from the aerial parts of Iranian *O. basilicum* were methyl chavicol (49.7%), linalool (10.7%), α-cadinol (5.9%), (Z)-β-farnesene (3.8%), and 1,8-cineole (3.5%). Conversely, further studies reported methyl chavicol or estragol as one of the main BEO constituents that instead resulted totally absent in the BEO analyzed in the present study, even if also sweet basil, the European type, contains both linalool and methylchavicol as the major constituents [30]. For example, linalool is the most abundant component in Serbian BEO (31.6%) followed by methyl chavicol (23.8%) [34], whereas the BEO tested in the present study derives from the cv. Genovese Gigante, the most used in the production of a typical Italian sauce called “pesto” and shows linalool and eugenol as the main components [35].

### 3.2. BEO Antimicrobial Activity

The results reported in Table 3 show that *B. thermosphacta* D274, *E. faecalis* 226, are the strains more sensitive to BEO respect to the two antibiotics used as control. Instead against *C. maltaromaticum* 9P, *C. maltaromaticum* D1203, *E. coli* 32 and *S. salivarius* GM, BEO exhibits an antimicrobial activity higher than that of gentamicin but similar or lower than tetracycline. Conversely, for *E. faecalis* E21 and *H. alvei* 53M.

Moreover, the data obtained from disc diffusion method, followed by measurement of MIC, indicate that *E. coli* 32, *S. salivarius* GM, and *C. maltaromaticum* D1203 showed the lower values of MIC (1.25 mg/mL, respectively) (Table 4).

Therefore, BEO was shown to exhibit strong antimicrobial activity against all microorganisms tested, both Gram-positive and Gram-negative bacteria, in agreement with previous investigations [36], even though Gram-positive strains seem to be more sensitive to BEO [37,38,39]. Overall, the observed antimicrobial activity of BEO might be attributed to the high contents of linalool that possesses a stronger antimicrobial activity against Gram-positive bacteria than against Gram-negative bacteria [40,41].

### 3.3. BEO Microencapsulation and Antimicrobial Activity of BEOMC

**Figure 2:**
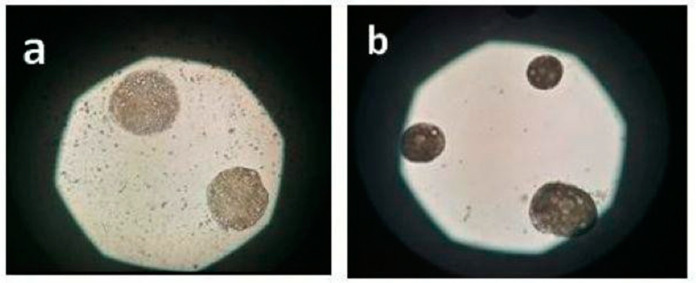
Optical microscopy image of basil essential oil microcapsules before (**a**) and after (**b**) washing with sterile water.

**Figure 3:**
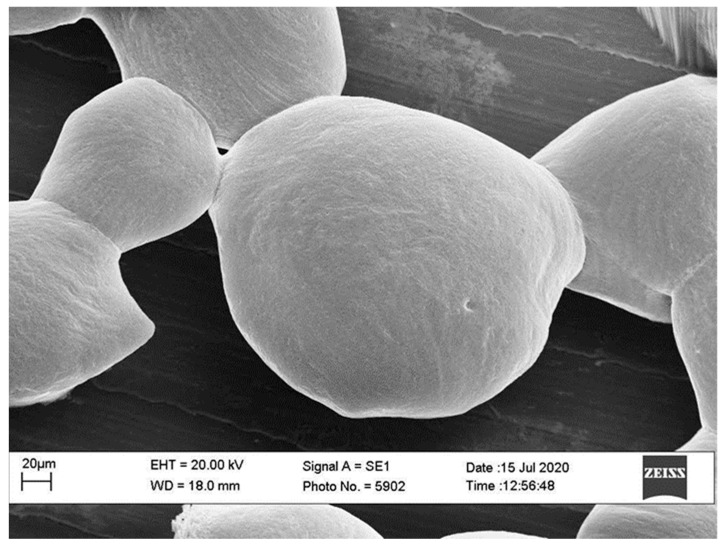
SEM image of basil essential oil microcapsules immediately after their production.

BEO was successfully microencapsulated by vibration technology with a process efficiency of about 87%. Results of size analysis of BEOMC showed a diameter range of 120–150 µm with more represented size in the range of 140–150 µm. Gap between nozzle size (120 µm) and BMCO size, as well as the size variability of BEOMC, is much probably due to the complexity and interaction of equipment parameters involved, as well explained by Chen et al. (2014) [17]. Figure 2 illustrates BEOMC optical microscopy image immediately after the microencapsulation (panel A) and before (a) and after (b) washing with sterile water to eliminate the oil outside the capsules, whereas Figure 3 shows the BEOMC SEM image. Images of Figure 2 show the presence of highly light refracting areas that likely are the droplets of BEO. Interestingly, after washing of BEOMC the presence of these areas seems decreasing letting thinking that part of BEO droplets were on the surface of the microcapsules before washing. On the other hand, we found, as up reported, that about 13% of BEO were not entrapped during the microencapsulation process. SEM image shows a smooth surface of BEOMC, in contrast to previous our results on alginate microcapsules containing bacterial cells [25], in which surface appeared rough. This result suggests that the surface morphology of alginate microcapsules could be affected by the nature of material is encapsulated. We hypothesize that materials like bacterial cells and nisin can chemically interact with alginate promoting perturbation of the polymer network, visible as roughness. On the contrary, components of BEO are much less reactive towards alginate.

**Figure 4:**
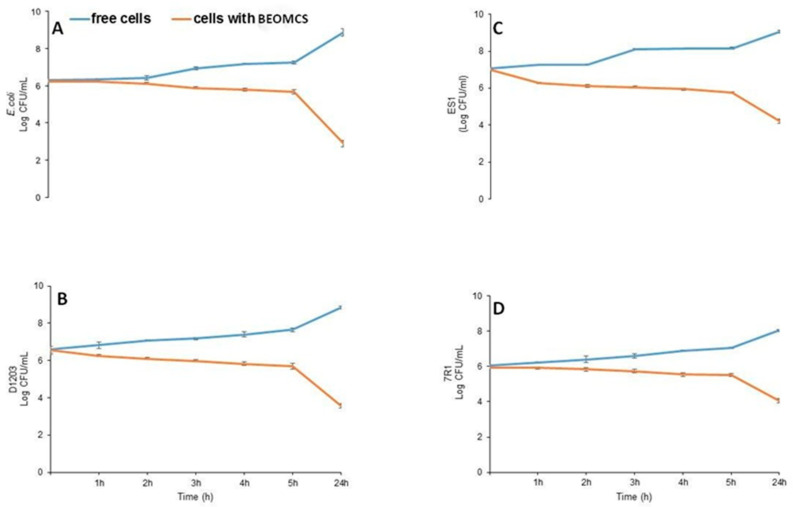
Viable counts (Log CFU/mL) of different microorganisms grown in contact with basil essential oil microcapsules. *E. coli* (**A**), D1203 (**B**), ES1 (**C**), 7R1 (**D**).

The study of antimicrobial activity of BEOMC showed that the inhibitory effect of BEO against all microorganisms tested was fully preserved. Total viable counts of the strains tested in contact with BEOMC are reported in Figure 4. All data indicate that the bacterial load decreased in the presence of microcapsules. More in particular, the number of CFU/mL of *E. coli 32* remained constant for the first 2 h of contact with BEOMC, starting then to decrease and reaching a dramatic reduction after 24 h (Figure 4, panel A). A similar behavior, even though quantitatively less marked, was observed by testing *C. maltaromaticum* D1203 (Figure 4, panel B), *S. xylosus* ES1 (Figure 4, panel C) and *B. thermosphacta* 7R1 (Figure 4, panel D). Supplementary Materials Figure S1 reports viability images of *E. coli* using fluorescence microscopy. Panel a of Figure S1 shows that all cells were green-stained, and thus alive, in the absence of microparticles. In contrast, when *E. coli* cells were in contact with BEOMCs, the red staining indicated the beginning of their viability loss after 1 and 5 h of contact (Figure S1, panel b and c, respectively) whereas, after 24 h, the almost the entire bacterial population resulted damaged (Figure S1, panel d). The lethal effect of BEOMCs has been attributed to the high level of linalool, able to produce membrane and cell wall damage, causing leakage of macromolecules and cell lysis. These results are in agreement with previous studies demonstrating that BEO microencapsulated by spray drying decreased initial population of *E. coli 32* from 5 Log CFU/mL to 2.9 Log CFU/mL after 6 h of incubation, and that the antimicrobial effect was due to compounds present in the EO able to alter the cytoplasmic membrane, allowing the leakage of intracellular constituents, because of their hydrophobic characteristics [42,43,44,45].

### 3.4. Preparation and Physicochemical Properties of CH-Based Films Grafted with BEOMC

**Figure 5:**
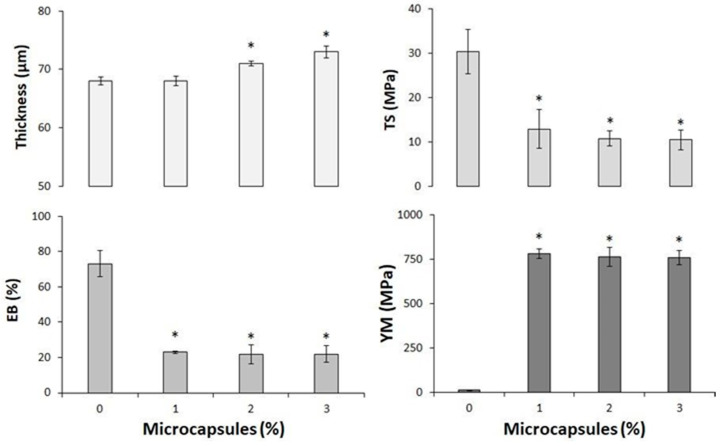
Thickness and mechanical properties (TS, EB, and YM) of CH films, obtained at pH 4.5 in the presence of 30% glycerol and different concentrations of basil essential oil microcapsules. The asterisks indicate the values significantly different at *p* < 0.05 from those obtained with CH films prepared in the absence of basil essential oil microcapsules.

CH films were prepared both in the presence and absence of BEOMC and some of their main physicochemical features were investigated. Firstly, the presence of microcapsules was clearly visible into the film to the naked eye (Figure S2). Figure 5 reports the changes observed in thickness, TS, EB, and YM of the CH films grafted with different BEOMC concentrations. Adding 2 and 3% of BEO containing microcapsules to CH FFS led to produce films exhibiting a significantly increased thickness (71.0 ± 0.4 and 73.0 ± 1.0, respectively), compared to that of control films (68.0 ± 0.7). The presence of BEOMC, increasing the free volume inside the CH network and, consequently, enhancing the distance between the CH chains into the polymeric matrix, results at the end in the production of a relatively thicker material [46,47]. Conversely, the TS of CH film containing 1, 2, and 3% of BEOMC was (13.0 ± 4.3 MPa; 10.8 ± 1.7 MPa and 10.5 ± 2.3 MPa, respectively) and EB (23.0 ± 0.7%; 22.0 ± 5.4% and 22.0 ± 4.8%, respectively) were found to be significantly lower in comparison with the control films were the TS and EB was (30.5 ± 5.0 MPa and EB 73.2 ± 7.3% respectively). These results are agreement with those recently obtained by Jang et al. (2020) with CH films containing encapsulated lemon EO. Whereas the YM was markedly increased in the presence of 1, 2, and 3% of BEOMC (780.0 ± 28.0 MPa; 763.0 ± 55.0 MPa and 758.0 ± 42.0 MPa, respectively) comparing to CH film alone (11.3 ± 1.7 MPa), we concluded that the presence of microcapsules gave rise to heterogeneous film networks with a discontinuous microstructure due to a rearrangement of the CH chains into the matrix [47,48,49].

**Figure 6:**
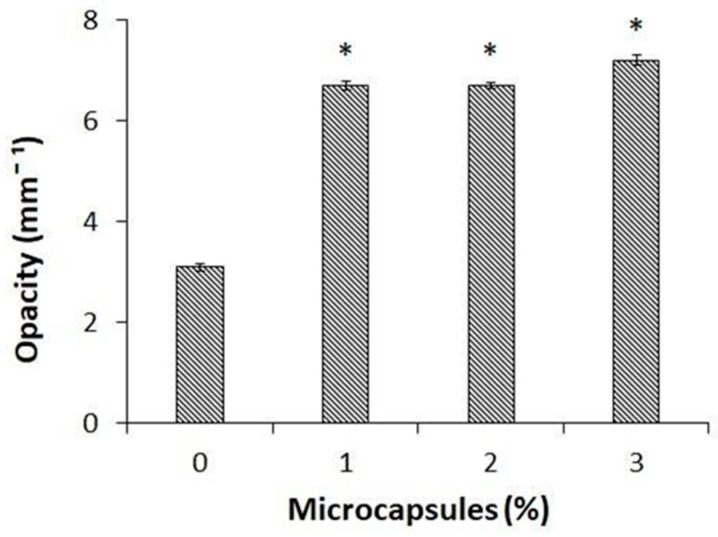
Effect of different concentrations of basil essential oil microcapsules on the opacity of CH films obtained at pH 4.5 in the presence of 30% glycerol. The asterisks indicate the values significantly different at *p* < 0.05 from those obtained with CH films prepared in the absence of basil essential oil microcapsules.

Since the obtained BEO encapsulated materials might be used to wrap food products, the appearance of the packaging material represents a very critical parameter for the consumers. Thus, film opacity was evaluated by detecting the light transmission at 600 nm through the CH films containing different amounts of BEOMCs. Figure 6 shows that film opacity was significantly increased, more than doubling, in the films containing even only 1% (6.7 ± 0.08 mm^−1^) of microcapsules compared to the control samples (3.1 ± 0.07 mm^−1^), confirming previous studies that have demonstrated that film transparency decreased in all the films prepared in the presence of either nanoparticles or microcapsules [29,50,51].

### 3.5. Antimicrobial Activity of CH Films Grafted with BEOMC

**Figure 7:**
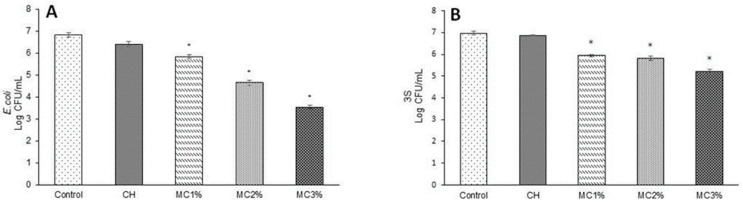
Antimicrobial activity of chitosan (CH) films grafted with different amounts of basil essential oil microcapsules (MC) against *E. coli* Panel (**A**) and *S. saprophyticus* 3S Panel (**B**). The asterisks indicate the values significantly different at *p* < 0.05 from those obtained without film addition (control) or with CH films prepared in the absence of basil essential oil MC.

The antimicrobial activity of CH edible films grafted or not with microencapsulated BEO was investigated against Gram-positive *S. saprophyticus* 3S and Gram-negative *E. coli* 32 bacteria. The results reported in Figure 7 show that CH-based films prepared in the absence of BEOMC did not exhibit antimicrobial effects against the evaluated strains in comparison with the control samples. Conversely, CH films containing increasing concentrations of BEOMC were able to significantly reduce cell viability of both strains, films containing 3% of microcapsules being able to reduce the total initial population of *E. coli 32* by 3 Log (Figure 7, panel A) and that of *S. saprophyticus* 3S by 2 Log (Figure 7, panel B). Although CH is known to possess antimicrobial activity, it was shown to be inactive against a serials of pathogenic and spoilage bacteria [52,53,54], probably because of different factors including experimental conditions (concentrations, pH, type of microorganism, and neighboring components) as well as its molecular properties (molecular weight, degree of deacetylation, and original source) [55]. In general, however, incorporation of the EOs conferred or enhanced antibacterial efficiency of CH films against different spoilage microorganisms and food-borne pathogens [56,57,58]. In this respect, Cristani et al. (2017) [59], reported that the observed antimicrobial action can be attributed to the EO content in terpenes that affect the permeability and other functions of the bacterial membranes. Monoterpenes would increase the concentration of lipidic peroxides, such as hydroxyl, alkoxyl and alkoperoxyl radicals, causing cell death. In the present study this effect could be due to specific chemical components present in BEO [36], which could be responsible for cell membrane disruption thereby leading to cell death.

### 3.6. Cooked Ham Wrapped with BEOMC Containing CH Films

**Figure 8:**
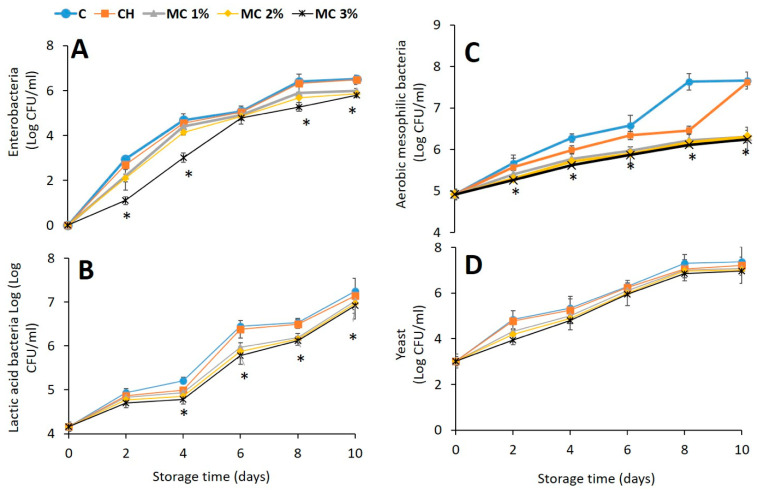
Inhibitory effect on bacterial growth of different concentrations of basil essential oil microcapsules (MC) present in chitosan (CH) films used to wrap cooked ham at different times of storage against Enterobacteria (**A**), Lactic acid bacteria (**B**), Aerobic mesophilic bacteria (**C**), and Yeast (**D**). The asterisks indicate the values significantly different at *p* < 0.05 from those obtained with unwrapped (**C**) or CH film-wrapped cooked ham samples in the absence of basil essential oil MC.

To investigate the possible preservative effect of food packaging by CH films containing BEOMC, microbiological analyses at different times of refrigerated storage of cooked ham samples, wrapped with films containing different amounts of microcapsules, were carried out. Population of enterobacteria, lactic acid bacteria, aerobic mesophilic bacteria, and yeasts was taken into account. The results reported in Figure 8 indicate an almost general similar trend for all microbial populations examined (Figure 8, panels A, B, C and D). In fact, with the exception of yeast (Figure 8, panel D) resulting unaffected, the counting of all the viable cells of both controls (unwrapped and CH film-wrapped samples) was always higher compared to that detected, at the same time of cooked ham storage, with the food samples wrapped by CH films containing BEOMC. More in particular, the food wrapping with CH films containing only 1% of microcapsules was effective in reducing microbial counts with the maximum effect observed on the aerobic mesophilic bacteria at 8–10 days when the presence of BEOMC decreased the cell count by 3 log CFU/mL (Figure 8, panel C). Similar results were previously obtained by using thyme and oregano EOs [60,61,62,63].

**Figure 9:**
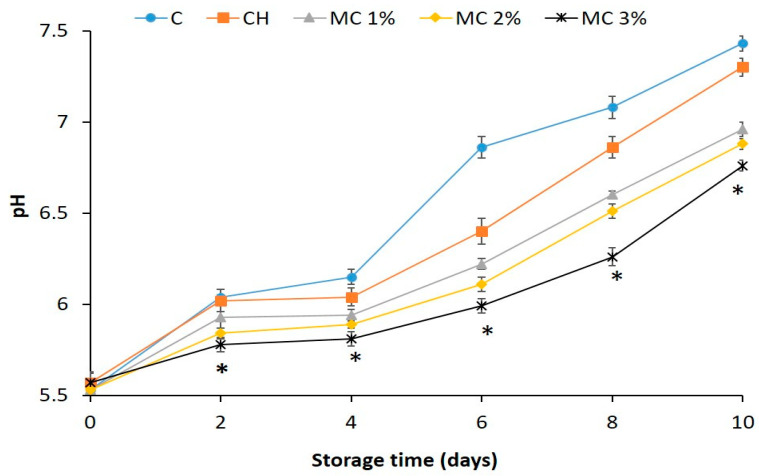
pH increase in cooked ham samples unwrapped (control, C) and wrapped with films of chitosan (CH) alone or with CH films containing different concentrations of basil essential oil microcapsules (MC) at different times of storage. The asterisks indicate the values significantly different at *p* < 0.05 from those obtained with unwrapped (C) or CH film wrapped cooked ham samples in the absence of MC.

Finally, the variation of pH value of both unwrapped and differently wrapped cooked ham samples was investigated during the food storage. Figure 9 shows that the pH of all cooked ham samples increased during the storage, but the pH increase observed in the samples wrapped with CH films containing BEOMC (Figure 9) was lower than that of both controls at all times of storage. Similar results have been reported by analyzing chicken thigh [64] and EO-packaged poultry meat [65]. In this respect, Silva et al. (2002) [66], suggested that the increase in pH during food storage is related to the formation and accumulation of amines and ammonia probably due to an increase of the lactic acid bacteria population.

## 4. Conclusions

The present study demonstrated that BEO have a marked antimicrobial activity, it could be attributed to its high content of linalool, both in its free form and when it is microencapsulated, as well as the BEOMC were incorporated in CH films. Moreover, the wrapping of cooked ham samples with CH films containing BEOMC was found to decrease mainly the growth of aerobic mesophilic bacteria and the enhancement of food pH during its storage. Therefore, these findings suggest that CH films containing BEOMC might be used as preservative active packaging to enhance the safety and prolong the shelf life of different kinds of food. However, other issues need to be addressed in future works such as the sensorial characteristics (flavor, color, and odor).

## Figures and Tables

**Table 1:** Source and optimal growth conditions of microorganisms.

Gram	Microorganism	Source	Growth Condition
Positive	*Brochothrix thermosphacta* 7R1	Meat	TSB 24 h at 20 °C
	*Brochothrix thermosphacta* D274	Meat	TSB 24 h at 20 °C
	*Carnobacterium maltaromaticum* 9P	Meat	TSB 24 h at 20 °C
	*Carnobacterium maltaromaticum* D1203	Meat	TSB 24 h at 25 °C
	*Enterococcus faecalis* 226	Milk	TSB 24 h at 30 °C
	*Staphylococcus xylosus* ES1	Fermented meat	TSB 24 h at 37 °C
	*Staphylococcus saprophyticus* 3S	Fermented meat	TSB 24 h at 37 °C
	*Listeria innocua* 1770	Milk	TSB 24 h at 30 °C
	*Streptococcus salivarius* GM	Milk	TSB 24 h at 30 °C
Negative	*Hafnia alvei* 53M	Meat	TSB 24 h at 30 °C
	*Serratia proteamaculans* 20P	Meat	TSB 24 h at 30 °C
	*Escherichia coli* 32	Meat	TSB 24 h at 37 °C

**Table 2:** Basil essential oil (BEO) chemical composition *.

N.	Compound Name	%	KI ^a^	KI ^b^	Identification ^c^
1	Santolina triene	1.2	863	908	1,2
2	Artemisia triene	Traces	875	929	1,2
3	α-pinene	1.8	899	939	1,2,3
4	β-pinene	1.0	919	979	1,2,3
5	δ-3-Carene	Traces	939	1011	1,2
6	p-Cymene	0.1	948	1024	1,2,3
7	1,8-Cineole	9.6	953	1096	1,2,3
8	dehydro-sabina ketone	0.3	973	1120	1,2
9	neo-isopulegol	0.3	989	1148	1,2
10	iso-isopulegol	0.7	994	1159	1,2
11	Linalool	41.3	1033	1096	1,2,3
12	Terpinolene	0.1	1035	1088	1,2,3
13	(6Z)-Nonenal	0.1	1037	1097	1,2
14	iso-3-thujanol	0.2	1039	1138	1,2
15	neo-allo-ocimene	0.1	1048	1144	1,2
16	neo-iso-3-thujanol	Traces	1050	1151	1,2
17	iso-borneol	0.3	1058	1160	1,2
18	3-thujanol	Traces	1060	1168	1,2,3
19	thuj-3-en-10-al	0.2	1062	1184	1,2
20	cis-dihydrocarvone	0.1	1080	1192	1,2
21	trans-pulegol	0.6	1091	1214	1,2
22	cis-sabinene hydrate	1.4	1099	1221	1,2
23	(Z)-Anethole	3.2	1106	1252	1,2,3
24	isobornyl acetate	2.1	1189	1285	1,2
25	δ-elemene	0.2	1232	1338	1,2,3
26	trans-p-menth-6-en-2,8-diol	0.2	1240	1374	1,2
27	α-ylangene	0.1	1244	1375	1,2,3
28	(z)-isoeugenol	5.9	1259	1407	1,2
29	α-gurjunene	0.5	1269	1409	1,2
30	€-Caryophyllene	2.4	1288	1419	1,2,3
31	β-Ylangene	1.1	1304	1420	1,2,3
32	β-copaene	0.5	1314	1432	1,2,3
33	α-trans-bergamotene	4.6	1326	1434	1,2
34	Aromadendrene	0.3	1330	1441	1,2,3
35	α-humulene	0.8	1338	1454	1,2,3
36	allo-aromadendrene	1.1	1347	1460	1,2
37	cis-muurola-4-(14),5-diene	0.9	1365	1466	1,2
38	γ-gurjunene	0.5	1371	1477	1,2,3
39	γ-muurolene	0.8	1380	1479	1,2,3
40	Aristolochene	1.4	1391	1488	1,2
41	γ-himalachene	0.4	1396	1482	1,2
42	trans-muurola-4-(14),5-diene	2.8	1395	1493	1,2
43	cis-calamenene	0.3	1402	1529	1,2
44	δ-cadinene	0.4	1403	1523	1,2
45	10-epi-cubebol	0.1	1410		1,2
46	trans-cadina-1,4-diene	0.2	1416	1534	1,2
47	cis-muurol-5-en-4-β-ol	0.1	1435	1551	1,2
48	germacrene B	0.3	1444	1561	1,2,3
49	Spathulenol	1.0	1456	1578	1,2,3
50	cis-β—elemenone	0.4	1481	1589	1,2
51	1,10-di-epi-cubenol	1.2	1492	1619	1,2
52	1-epi-cubenol	4.8	1514	1628	1,2
	Total	97.8			
	Monoterpene hydrocarbons	3.1			
	Oxygenated monoterpenes	66.4			
	Sesquiterpene hydrocarbons	19.5			
	Oxygenated sesquiterpenes	7.6			
	Other	1.2			

* The compounds are listed according to their elution order on a HP-5MS column. ^a^: Linear retention index on a HP-5MS column; ^b^: Linear retention index on a HP Innowax column; ^c^: Identification method, 1 = linear retention index; 2 = identification based on the comparison of mass spectra; 3 = Co-injection with standard compounds.

**Table 3:** Antimicrobial activity of BEO compared to gentamicin and tetracycline *.

Scheme	Gentamicin	Tetracycline	BEO
*B. thermosphacta* 7R1	18.3 ± 1.5	19.3 ± 1.2	17.3 ± 1.1
*B. thermosphacta* D274	6.0 ± 0.0	8.7 ± 1.2	17.7 ± 0.6 ^a,b^
*C. maltaromaticum* 9P	6.0 ± 0.0	24.3 ± 1.2	11.7 ± 0.6 ^a^
*C. maltaromaticum* D1203	6.0 ± 0.0	22.3 ± 0.6	20.0 ± 1.0 ^a^
*E. coli* 32	14.7 ± 0.6	18.7 ± 1.2	20.7 ± 0.6 ^a^
*E. faecalis* 226	6.0 ± 0.0	9.0 ± 1.0	12.7 ± 0.6 ^a,b^
*E. faecalis* E21	6.0 ± 0.0	14.7 ± 0.6	11.3 ± 1.1 ^b^
*H. alvei* 53M	11.7 ± 1.5	9.6 ± 0.6	11.7 ± 0.6 ^b^
*L. innocua* 1770	25.3 ± 0.6	20.3 ± 1.5	16.3 ± 1.1
*S. proteamaculans* 20P	12.3 ± 0.6	24.3 ± 1.2	10.3 ± 0.6
*S. salivarius* GM	6.0 ± 0.0	18.7 ± 1.2	19.7 ± 0.6 ^a^
*S. saprophyticus* 3S	24.0 ± 1.0	29.0 ± 3.6	17.7 ± 0.4
*S. xylosus* ES1	19.3 ± 1.2	29.3 ± 1.2	18.0 ± 1.0

* Results are the mean of three tests ± standard deviation (SD) of the inhibition zone expressed in mm of diameter; ANOVA test vs. Gentamicin (^a^) or Tetracycline (^b^) *p <* 0.05.

**Table 4:** BEO minimal inhibitory concentration and minimal lethal concentration.

Strains	BEO	
	MIC (μL/mL)	MLC (μL/mL)
*C. maltaromaticum* D1203	1.25	2.50
*S. salivarius* GM	1.25	2.50
*S. saprophyticus* 3S	2.50	2.50
*E. coli* 32	1.25	1.25

## Supplementary Materials

The following are available online at https://www.mdpi.com/2304-8158/10/1/121/s1, Figure S1: Fluorescence microscopy viability images (100× magnification) of *E. coli* control culture (a) and in contact with basil essential oil microcapsules after 1 h (b), 5 h (c) and 24 h (d), Figure S2: CH film containing microcapsules of BEO.

## References

[1] Irkin R., Esmer O.K. (2015). Novel food packaging systems with natural antimicrobial agents. J. Food Sci. Technol..

[2] Suppakul P., Sonneveld K., Bigger S.W., Miltz J. (2011). Loss of AM additives from antimicrobial films during storage. J. Food Eng..

[3] Biji K.B., Ravishankar C.N., Mohan C.O., Srinivasa Gopal T.K. (2015). Smart packaging systems for food applications: A review. J. Food Sci. Technol..

[4] Li Z.-H., Cai M., Liu Y.-S., Sun P.-L., Luo S.-L. (2019). Antibacterial activity and mechanisms of essential oil from *Citrus medica* L. var. *sarcodactylis*. Molecules.

[5] Ch M., Naz S., Sharif A., Akram M., Saeed M. (2015). Biological and Pharmacological Properties of the Sweet Basil (*Ocimum basilicum*). Br. J. Pharm. Res..

[6] Murbach Teles Andrade B.F., Nunes Barbosa L., Da Silva Probst I., Fernandes Júnior A. (2014). Antimicrobial activity of essential oils. J. Essent. Oil Res..

[7] Gaio I., Saggiorato A.G., Treichel H., Cichoski A.J., Astolfi V., Cardoso R.I., Toniazzo G., Valduga E., Paroul N., Cansian R.L. (2015). Antibacterial activity of basil essential oil (*Ocimum basilicum* L.) in Italian-type sausage. J. Verbrauch. Lebensm..

[8] Embuscado M.E., Huber K.C. (2009). Edible Films and Coatings for Food Applications.

[9] Kausadikara S., Gadhaveb A.D., Waghmareb J. (2015). Microencapsulation of lemon oil by spray drying and its applicationin flavour tea. Adv. Appl. Sci. Res..

[10] Gibbs B.F., Kermasha S., Alli I., Mulligan C.N. (1999). Encapsulation in the food industry: A review. Int. J. Food Sci. Nutr..

[11] Nemethova V. (2015). Vibration technology for microencapsulation: The restrictive role of viscosity. J. Bioprocess. Biotech..

[12] Shahidi F., Han X.Q. (1993). Encapsulation of food ingredients. Crit. Rev. Food Sci. Nutr..

[13] Ozdemir M., Kemerli T. (2016). Innovative applications of micro and nanoencapsulation in food packaging. Encapsulation and Controlled Release Technologies in Food Systems.

[14] Quesada J., Sendra E., Navarro C., Sayas-Barberá E. (2016). Antimicrobial active packaging including chitosan films with *Thymus vulgaris L.* essential oil for ready-to-eat meat. Foods.

[15] Almeida A.P., Rodríguez-Rojo S., Serra A.T., Vila-Real H., Simplicio A.L., Delgadilho I., Beirão Da Costa S., Beirão Da Costa L., Nogueira I.D., Duarte C.M.M. (2013). Microencapsulation of oregano essential oil in starch-based materials using supercritical fluid technology. Innov. Food Sci. Emerg. Technol..

[16] Rodea-González D.A., Cruz-Olivares J., Román-Guerrero A., Rodríguez-Huezo M.E., Vernon-Carter E.J., Pérez-Alonso C. (2012). Spray-dried encapsulation of chia essential oil (*Salvia hispanica* L.) in whey protein concentrate-polysaccharide matrices. J. Food Eng..

[17] Peng C., Zhao S.Q., Zhang J., Huang G.Y., Chen L.Y., Zhao F.Y. (2014). Chemical composition, antimicrobial property and microencapsulation of Mustard (*Sinapis alba*) seed essential oil by complex coacervation. Food Chem..

[18] Sabbah M., Di Pierro P., Cammarota M., Dell’Olmo E., Arciello A., Porta R. (2019). Development and properties of new chitosan-based films plasticized with spermidine and/or glycerol. Food Hydrocoll..

[19] Adams R.P. (2007). Identification of Essential Oil Components by Gas Chromatography/Mass Spectroscopy.

[20] Davies N.W. (1990). Gas chromatographic retention indices of monoterpenes and sesquiterpenes on methyl silicon and Carbowax 20M phases. J. Chromatogr. A.

[21] Goodner K.L. (2008). Practical retention index models of OV-101, DB-1, DB-5, and DB-Wax for flavor and fragrance compounds. LWT-Food Sci. Technol..

[22] Wiley Registry of Mass Spectral Data: WITH NIST Spectral Data CD-ROM: Inc. John Wiley & Sons: 9780470047866. https://www.bookdepository.com/Wiley-Registry-Mass-Spectral-Data-WITH-NIST-Spectral-Data-CD-ROM-Inc-John-Wiley-Sons/9780470047866.

[23] Amor G., Caputo L., La Storia A., De Feo V., Mauriello G., Fechtali T. (2019). Chemical composition and antimicrobial activity of *Artemisia herba-alba* and *Origanum majorana* essential oils from Morocco. Molecules.

[24] Sbayou H., Ababou B., Boukachabine K., Manresa A., Zerouali K., Amghar S. (2014). Chemical Composition and Antibacterial Activity of Artemisia herba-alba and Mentha pulegium Essential Oils. J. Life Sci..

[25] De Prisco A., Maresca D., Ongeng D., Mauriello G. (2015). Microencapsulation by vibrating technology of the probiotic strain *Lactobacillus reuteri* DSM 17938 to enhance its survival in foods and in gastrointestinal environment. LWT Food Sci. Technol..

[26] Ercolini D., La Storia A., Villani F., Mauriello G. (2006). Effect of a bacteriocin-activated polythene film on Listeria monocytogenes as evaluated by viable staining and epifluorescence microscopy. J. Appl. Microbiol..

[27] Di Pierro P., Chico B., Villalonga R., Mariniello L., Damiao A.E., Masi P., Porta R. (2006). Chitosan-whey protein edible films produced in the absence or presence of transglutaminase: Analysis of their mechanical and barrier properties. Biomacromolecules.

[28] ASTM D882-09 Standard Test Method for Tensile Properties of Thin Plastic Sheeting. https://www.astm.org/DATABASE.CART/HISTORICAL/D882-09.htm.

[29] Giosafatto C., Sabbah M., Al-Asmar A., Esposito M., Sanchez A., Villalonga Santana R., Cammarota M., Mariniello L., Di Pierro P., Porta R. (2019). Effect of Mesoporous Silica Nanoparticles on Glycerol-Plasticized Anionic and Cationic Polysaccharide Edible Films. Coatings.

[30] Filip S. (2017). Basil (*Ocimum basilicum* L.) a source of valuable phytonutrients. Int. J. Clin. Nutr. Diet..

[31] Milenković L., Stanojević J., Cvetković D., Stanojević L., Lalević D., Šunić L., Fallik E., Ilić Z.S. (2019). New technology in basil production with high essential oil yield and quality. Ind. Crops Prod..

[32] Olugbade T.A., Kolipha-Kamara M.I., Elusiyan C.A., Onawunmi G.O., Ogundaini A.O. (2017). Essential Oil Chemotypes of Three Ocimum Species Found in Sierra Leone and Nigeria. Med. Aromat. Plants.

[33] Ghasemi Pirbalouti A., Malekpoor F., Salimi A. (2017). Chemical composition and yield of essential oil from two Iranian species of basil (*Ocimum ciliatum* and *Ocimum basilicum*). Trends Phytochem. Res..

[34] Stanojevic L.P., Marjanovic-Balaban Z.R., Kalaba V.D., Stanojevic J.S., Cvetkovic D.J., Cakic M.D. (2017). Chemical composition, antioxidant and antimicrobial activity of basil (*Ocimum basilicum* L.) essential oil. J. Essent. Oil-Bear. Plants.

[35] Miele M., Dondero R., Ciarallo G., Mazzei M. (2001). Methyleugenol in *Ocimum basilicum* L. cv. Genovese Gigante. J. Agric. Food Chem..

[36] Suppakul P., Miltz J., Sonneveld K., Bigger S.W. (2003). Antimicrobial properties of basil and its possible application in food packaging. J. Agric. Food Chem..

[37] Dhifi W., Bellili S., Jazi S., Bahloul N., Mnif W. (2016). Essential Oils’ Chemical Characterization and Investigation of Some Biological Activities: A Critical Review. Medicines.

[38] Hussain A.I., Anwar F., Hussain Sherazi S.T., Przybylski R. (2008). Chemical composition, antioxidant and antimicrobial activities of basil (*Ocimum basilicum*) essential oils depends on seasonal variations. Food Chem..

[39] Vanin A.B., Orlando T., Piazza S.P., Puton B.M.S., Cansian R.L., Oliveira D., Paroul N. (2014). Antimicrobial and antioxidant activities of clove essential oil and eugenyl acetate produced by enzymatic esterification. Appl. Biochem. Biotechnol..

[40] Ebrahim Sajjadi S. (2006). Analysis of the essential oils of two cultivated basil (*Ocimum basilicum* L.) from Iran. J. Pharm. Sci..

[41] Soković M., Van Griensven L.J.L.D. (2006). Antimicrobial activity of essential oils and their components against the three major pathogens of the cultivated button mushroom, *Agaricus bisporus*. Eur. J. Plant Pathol..

[42] Bagamboula C.F., Uyttendaele M., Debevere J. (2004). Inhibitory effect of thyme and basil essential oils, carvacrol, thymol, estragol, linalool and p-cymene towards Shigella sonnei and S. flexneri. Food Microbiol..

[43] Burt S. (2004). Essential oils: Their antibacterial properties and potential applications in foods—A review. Int. J. Food Microbiol..

[44] Di Pasqua R., Betts G., Hoskins N., Edwards M., Ercolini D., Mauriello G. (2007). Membrane toxicity of antimicrobial compounds from essential oils. J. Agric. Food Chem..

[45] Hernández-Hernández E., Regalado-González C., Vázquez-Landaverde P., Guerrero-Legarreta I., García-Almendárez B.E. (2014). Microencapsulation, chemical characterization, and antimicrobial activity of Mexican (*Lippia graveolens* H.B.K.) and European (*Origanum vulgare* L.) oregano essential oils. Sci. World J..

[46] Alves V.L.C.D., Rico B.P.M., Cruz R.M.S., Vicente A.A., Khmelinskii I., Vieira M.C. (2018). Preparation and characterization of a chitosan film with grape seed extract-carvacrol microcapsules and its effect on the shelf-life of refrigerated Salmon (*Salmo salar*). LWT Food Sci. Technol..

[47] Jiang Y., Lan W., Sameen D.E., Ahmed S., Qin W., Zhang Q., Chen H., Dai J., He L., Liu Y. (2020). Preparation and characterization of grass carp collagen-chitosan-lemon essential oil composite films for application as food packaging. Int. J. Biol. Macromol..

[48] Rodsamran P., Sothornvit R. (2018). Microencapsulation of Thai rice grass (O. Sativa cv. Khao Dawk Mali 105) extract incorporated to form bioactive carboxymethyl cellulose edible film. Food Chem..

[49] Li Y., Tang J.P., Chen D.R., Fu C.Y., Wang P., Li Z., Wei W., Li H., Dong W.Q. (2013). The use of albendazole and diammonium glycyrrhizinate in the treatment of eosinophilic meningitis in mice infected with Angiostrongylus cantonensis. J. Helminthol..

[50] Alarcón-Moyano J.K., Bustos R.O., Herrera M.L., Matiacevich S.B. (2017). Alginate edible films containing microencapsulated lemongrass oil or citral: Effect of encapsulating agent and storage time on physical and antimicrobial properties. J. Food Sci. Technol..

[51] Cai C., Ma R., Duan M., Deng Y., Liu T., Lu D. (2020). Effect of starch film containing thyme essential oil microcapsules on physicochemical activity of mango. LWT.

[52] Hosseini M.H., Razavi S.H., Mousavi M.A. (2009). Antimicrobial, physical and mechanical properties of chitosan-based films incorporated with thyme, clove and cinnamon essential oils. J. Food Process. Preserv..

[53] Ojagh S.M., Rezaei M., Razavi S.H., Hosseini S.M.H. (2010). Development and evaluation of a novel biodegradable film made from chitosan and cinnamon essential oil with low affinity toward water. Food Chem..

[54] Pelissari F.M., Grossmann M.V.E., Yamashita F., Pined E.A.G. (2009). Antimicrobial, mechanical, and barrier properties of cassava starch-chitosan films incorporated with oregano essential oil. J. Agric. Food Chem..

[55] Hosseinnejad M., Jafari S.M. (2016). Evaluation of different factors affecting antimicrobial properties of chitosan. Int. J. Biol. Macromol..

[56] López-Mata M.A., Ruiz-Cruz S., Silva-Beltrán N.P., Ornelas-Paz J.D.J., Zamudio-Flores P.B., Burruel-Ibarra S.E. (2013). Physicochemical, antimicrobial and antioxidant properties of chitosan films incorporated with carvacrol. Molecules.

[57] Raphaël K.J., Meimandipour A. (2017). Antimicrobial activity of chitosan film forming solution enriched with essential oils; an in vitro assay. Iran. J. Biotechnol..

[58] Yuan G., Chen X., Li D. (2016). Chitosan films and coatings containing essential oils: The antioxidant and antimicrobial activity, and application in food systems. Food Res. Int..

[59] Cristani M., D’Arrigo M., Mandalari G., Castelli F., Sarpietro M.G., Micieli D., Venuti V., Bisignano G., Saija A., Trombetta D. (2007). Interaction of four monoterpenes contained in essential oils with model membranes: Implications for their antibacterial activity. J. Agric. Food Chem..

[60] Emiroǧlu Z.K., Yemiş G.P., Coşkun B.K., Candoǧan K. (2010). Antimicrobial activity of soy edible films incorporated with thyme and oregano essential oils on fresh ground beef patties. Meat. Sci..

[61] Wen Z., You X., Jiang L., Liu B., Zheng Z., Pu Y., Cheng B. (2011). Liposomal incorporation of rose essential oil by a supercritical process. Flavour Fragr. J..

[62] Petrou S., Tsiraki M., Giatrakou V., Savvaidis I.N. (2012). Chitosan dipping or oregano oil treatments, singly or combined on modified atmosphere packaged chicken breast meat. Int. J. Food Microbiol..

[63] Ruiz-Navajas Y., Viuda-Martos M., Sendra E., Perez-Alvarez J.A., Fernández-López J. (2013). In vitro antibacterial and antioxidant properties of chitosan edible films incorporated with *Thymus moroderi* or *Thymus piperella* essential oils. Food Control.

[64] Silva C.M.G., Glória M.B.A. (2002). Bioactive amines in chicken breast and thigh after slaughter and during storage at 4 ± 1 °C and in chicken-based meat products. Food Chem..

[65] Oral N., Vatansever L., Sezer Ç., Aydin B., Güven A., Gülmez M., Başer K.H.C., Kürkçüoǧlu M. (2009). Effect of absorbent pads containing oregano essential oil on the shelf life extension of overwrap packed chicken drumsticks stored at four degrees Celsius. Poult. Sci..

[66] Silva C.M.G., Glória M.B.A. (2002). Biogenic Amine Sources in Cooked Cured Shoulder Pork. Food Chem..

