# Supplementary material for: Basil Essential Oil: Composition, Antimicrobial Properties, and Microencapsulation to Produce Active Chitosan Films for Food Packaging
Source: Foods. 2021 Jan 8;10(1):121. doi: 10.3390/foods10010121 (PMC7827191; doi:10.3390/foods10010121)
Supplement: Supplementary file 1 [file foods-10-00121-s001.pdf]

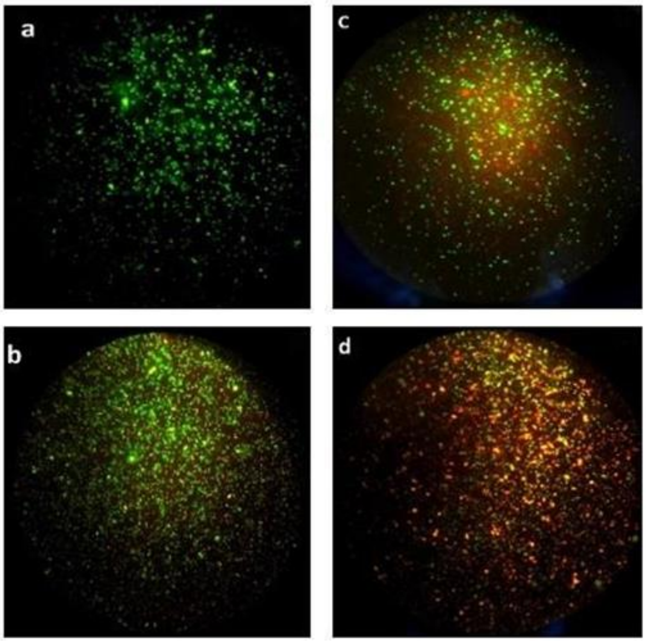

Figure S1. Fluorescence microscopy viability images (100 X magnification) of *E. coli* control culture (a) and in contact with basil essential oil microcapsules after 1 h (b), 5 h (c) and 24 h (d).

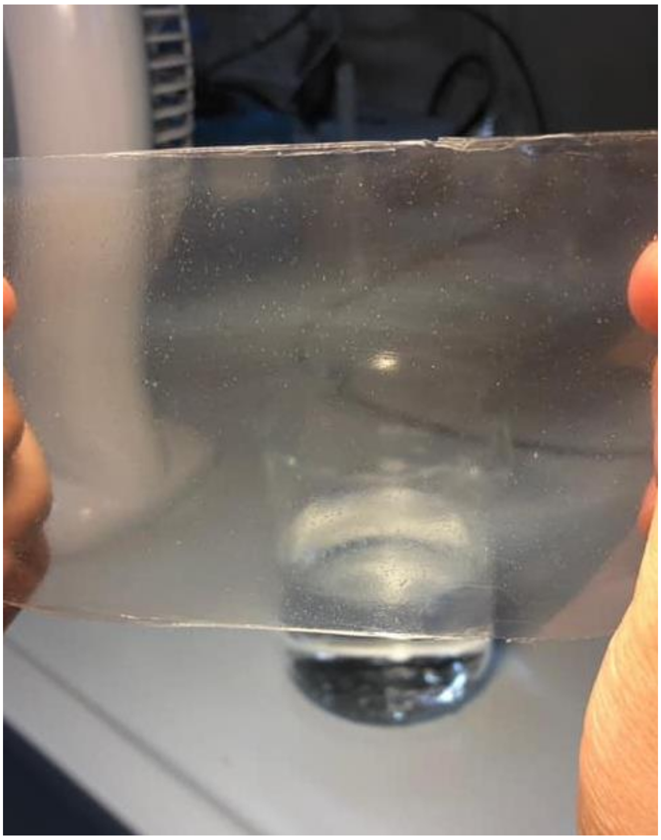

Figure S2. CH film containing microcapsules of BEO.
